# Probiotic *Bacillus* Alleviates Oxidative Stress-Induced Liver Injury by Modulating Gut-Liver Axis in a Rat Model

**DOI:** 10.3390/antiox11020291

**Published:** 2022-01-31

**Authors:** Yanping Wu, Baikui Wang, Li Tang, Yuanhao Zhou, Qi Wang, Li Gong, Jiajia Ni, Weifen Li

**Affiliations:** 1 Key Laboratory of Molecular Animal Nutrition of the Ministry of Education, College of Animal Sciences, Zhejiang University, Hangzhou 310058, China; ypwu@zafu.edu.cn (Y.P.); wangbaikui@zju.edu.cn (B.W.); 11717015@zju.edu.cn (L.T.); 12017005@zju.edu.cn (Y.Z.); 12117010@zju.edu.cn (Q.W.); 2 College of Animal Science and Technology, College of Veterinary Medicine, Zhejiang Agricultural and Forestry University, Hangzhou 311300, China; 3 School of Life Science and Engineering, Foshan University, Foshan 528011, China; 4 Research and Development Center, Guangdong Meilikang Bio-Science Ltd., Dongguan 523808, China

**Keywords:** *Bacillus*, rats, oxidative stress, liver injury, gut microbiota

## Abstract

Emerging evidence suggests a key role of gut microbiota in maintaining liver functions through modulating the gut–liver axis. In this study, we investigated whether microbiota alteration mediated by probiotic *Bacillus* was involved in alleviating oxidative stress- induced liver injury. Sprague–Dawley rats were orally administered *Bacillus* SC06 or SC08 for a 24-day period and thereafter intraperitoneally injected diquat (DQ) to induce oxidative stress. Results showed that *Bacillus*, particularly SC06 significantly inhibited hepatic injuries, as evidenced by the alleviated damaged liver structure, the decreased levels of ALT, AST, ALP and LDH, and the suppressed mitochondrial dysfunction. SC06 pretreatment markedly enhanced the liver antioxidant capacity by decreasing MDA and p47, and increasing T-AOC, SOD and HO-1.16S rRNA sequencing analysis revealed that DQ significantly changed the diversities and composition of gut microbiota, whereas *Bacillus* pretreatments could attenuate gut dysbiosis. Pearson’s correlation analysis showed that AST and MDA exerted a positive correlation with the opportunistic pathogenic genera and species (*Escherichia* and *Shigella*), and negatively correlated with the potential probiotics (*Lactobacillus*), while SOD exerted a reverse trend. The microbial metagenomic analysis demonstrated that *Bacillus*, particularly SC06 markedly suppress the metabolic pathways such as carbohydrate metabolism, lipid metabolism, amino acid metabolism and metabolism of cofactors and vitamins. Furthermore, SC06 decreased the gene abundance of the pathways mediating bacterial replication, secretion and pathogenicity. Taken together, *Bacillus* SC06 alleviates oxidative stress-induced liver injuries via optimizing the composition, metabolic pathways and pathogenic replication and secretion of gut microbiota. These findings elucidate the mechanisms of probiotics in alleviating oxidative stress and provide a promising strategy for preventing liver diseases by targeting gut microbiota.

## 1. Introduction

Oxidative stress occurs when the balance between the intracellular levels of reactive oxygen species (ROS) and the antioxidant defense system is disturbed, resulting in cellular damage by mediating three main reactions, namely lipid peroxidation, oxidation of proteins and nucleic acid damage [1]. A lot of risks like drugs, alcohol, obesity, pathogens and environmental stimuli can induce ROS generation [2,3]. Diquat [(1,1′-ethylene-2,2′-dipyridylium, DQ)] is a potent prooxidant that has been widely used both in vivo and in vitro studies for oxidative stress [4,5]. Liver is the main targeted organ, in which DQ utilizes molecular oxygen to produce ROS and eventually induces acute oxidative injuries [6]. Upon oxidative stress, the mitochondria and peroxisomes in Parenchymal cells can produce massive ROS, contributing to the gene expression of liver fatty acid oxidation. Hepatic stellate cells, Kupffer and endothelial cells are also potentially sensitive and easily exposed to oxidative molecules [7]. It has been reported that in stellate cells, ROS- induced lipid peroxidation activates the inflammation and fibrogenesis, and ultimately causes liver fat accumulation [8]. Furthermore, ROS generation promotes hepatic insulin resistance, necro-inflammation and finally leads to hepatocyte apoptosis [9]. Thus, oxidative stress is believed to be the key factor in the initiation and progression of many liver diseases, such as alcoholic liver disease, non-alcoholic fatty liver disease (NAFLD) and chronic viral hepatitis [10].

Gut microbiota is a diverse ecosystem that inhabit the gastrointestinal tract. It comprises at least 10^13^–10^14^ microbial cells and over 2000 distinct species [11]. The main function of this microbiome is to provide the host with enhanced metabolic capabilities, modulate GI tissue development, and protect against pathogens and stimuli [12]. The relationship between gut microbiota and oxidative stress has been fully elucidated by Marciano et al. (2019) [13]. Gut epithelia and other cell types can be activated by the microbiota and microbial signals to generate ROS. This physiologically generated ROS serve as second messengers in multiple signal transduction pathways and participate in various cellular signaling. When this homeostasis is disturbed, ROS are over-accumulated and oxidative stress occurs. Thus, the interaction between oxidative stress and gut microbiota plays a pivotal role in health and disease. The liver is at the crossroad between portal blood flow from intestinal circulation and peripheral organs, making it in close interplay with the intestinal tract which is termed “gut-liver axis” [14]. This axis offers liver specific interacts with a substantial amount of microbiota derived signals [15]. Emerging evidence suggests a key role of gut microbiota in the modulation of gut–liver axis health [16]. Metabolites of intestinal bacteria provide essential nutrients (e.g., vitamins and short-chain fatty acids) to maintain liver health, while a small portion of bacteria compounds delivered to the liver can mildly trigger immune responses [17]. When the intestinal microbiota is abnormal, pathogens or toxic products can reach the liver via the portal vein, which may induce inflammation, oxidative stress and lead to the progression of liver diseases [15,18]. Nevertheless, a healthy commensal microbiota is hepatoprotective and can prevent liver damages. Studies found that following liver injuries, products from the microbiota were involved in the protection and maintenance of liver homeostasis; the microbial-derived indole-3-propionic acid could provide hepatic protection from oxidative stress [19]. Therefore, maintaining a balanced microbiota signifies a promising strategy to alleviate liver injuries associated with oxidative stress.

Probiotics are defined as live microorganisms which, when administrated in adequate amounts, confer a promoting property to the host [20]. Their modulation on gut microbiota has been well recognized [21]. Recent studies have showed that probiotics such as *Lactobacillus johnsonii* and *Lactobacillus plantarum* can optimize the composition of gut microbiota to attenuate oxidative injuries in various models of oxidative stress [22,23]. Although alteration of gut microbiota has been reported to be a useful integrative treatment of chronic liver diseases [24,25], few studies have explored the protective effect of probiotics on oxidative stress-induced liver injuries and the underlying mechanisms remain unknown. Our previous results showed that *Bacillus amyloliquefaciens* SC06 and *Bacillus licheniformis* SC08 could alleviate oxidative stress-induced intestinal disorders and apoptosis [26]. On this basis, this study aimed to integrate the 16S rRNA sequencing and metagenomic analysis to clarify the mechanisms of the two strains in attenuating DQ-induced oxidative damages of liver in a rat model.

## 2. Materials and Methods

### 2.1. Bacteria Culture

Probiotic *Bacillus amyloliquefaciens* SC06 was preserved at the China Center for Type Culture Collection (CCTCC, No: M2012280), while *Bacillus licheniformis* SC08 was isolated from a commercial capsule [26]. These two strains were both grown overnight in Luria-Bertani (LB) broth at 37 °C. Bacteria were then washed, diluted to 10^7^ cfu/mL in 0.9% saline and prepared for rat oral administration.

### 2.2. Animal Experimental Design

All animal studies were carried out in accordance with the guidelines for the Institutional Animal Care and Use Committee of Zhejiang University (Ethical protocol code: ZJU20160416). Sixty 4-week-old male Sprague–Dawley rats were obtained from Slac Laboratory Animal Co., Ltd. (Shanghai, China). Rats were fed with a basal diet and housed in an environmentally controlled room. After a week of acclimatization, sixty rats were divided into three groups (*n* = 20 each) and orally administered 1 mL of 0.9% saline or 10^7^ cfu/mL SC06 or SC08 every day. After 24 days, half of the rats in each group were intraperitoneally injected with 12 mg/kg BW DQ (Sigma, St. Louis, MO, USA) in 0.9% saline, while the remaining rats received an equivalent volume of 0.9% saline. Animals were sacrificed by anesthesia 48 h after DQ injection. Blood, liver and intestinal samples were collected and stored at −80 °C until use.

### 2.3. Analysis of Biochemical Parameters

To assess the degree of liver injury, serum was collected to determine the activities of ALT, AST, ALP, and LDH using a biochemical autoanalyzer (7180, HITACHI, Tokyo, Japan). Oxidative stress indices including MDA, T-AOC, CAT, SOD and GSH-Px in liver samples were measured according to the manufacturer’s instructions (Jiancheng Bioengineering Institute, Nanjing, China).

### 2.4. Morphology Analysis

A small portion of fresh liver was immediately fixed in 4% paraformaldehyde PBS solution after rat sacrifice and embedded in paraffin. The tissue blocks were then sliced, mounted on glass slides and stained with hematoxylin and eosin. Images of each sample were obtained using an Olympus Microsystem (Tokyo, Japan). For transmission electron microscopy (TEM) analysis, liver samples were immediately fixed in 2.5% glutaraldehyde PBS solution and then fixed in 1% OsO_4_ buffer. After dehydration and infiltration, liver pellet was embedded in spurr resin and sectioned in LEICA EM UC7 ultratome. Sections were then stained by uranyl acetate and alkaline lead citrate, respectively, and observed in a Hitachi Model H-7650 TEM. For detecting ROS production, liver specimens were immediately stored at −80 °C. The frozen sections were then sliced into pieces and stained with Dihydroethidium (DHE, Sigma-Aldrich, St. Louis, MO, USA) at 37 °C for 30 min. After being washed with PBS three times, samples were sealed and visualized by an Olympus fluorescence microscope.

### 2.5. Detection of ΔΨm

The mitochondrial membrane potential (ΔΨm) was determined using a JC-1 probe kit (Beyotime, Shanghai, China). Firstly, a small section (50 mg) of liver was excised and weighted immediately after rat sacrifice. Liver mitochondria was obtained by a tissue mitochondria extraction kit (Beyotime, Shanghai, China). Thereafter, the purified mitochondria were stained with a 1× JC-1 working solution, and samples were detected by a fluorescence microplate. ΔΨm was calculated as the ratio of red fluorescence (JC-1 aggregates) to green fluorescence (JC-1 monomers).

### 2.6. Western Blot

Rat liver samples were homogenized and lysed in RIPA buffer (Beyotime, Shanghai, China) containing 1% PMSF. After denaturation, equal amounts of protein were loaded onto SDS-polyacrylamide gel, and then transferred to polyvinylidene difluoride membranes (Roche). Membranes were blocked with 5% non-fat milk in TBS at room temperature for 2 h, and incubated with primary antibodies overnight at 4 °C. The primary antibodies, including phospho-Nrf2, Nrf2, Keap1, and cytochrome C, were obtained from Cell Signal Technologies (Danvers, MA, USA). Antibodies targeting Heme Oxygenase 1 (HO-1) and P47^phox^ were from Abcam (Cambridge, MA, USA), while β-actin, HRP-conjugated anti-mouse IgG, and HRP-conjugate anti-rabbit IgG were from Beyotime (Shanghai, China). After incubating with secondary antibody for 1 h at room temperature, the immunoreactive bands were detected using an ECL system (Tanon, Shanghai, China). The relative band density was determined by Image J software. 

### 2.7. 16S rRNA-Based Microbiota Analysis

Genomic DNA was extracted using the QIAamp DNA Stool Mini Kit (QIAGEN, Hilden, Germany) according to the manufacturer’s protocol. Library preparation and 16S rRNA NGS were performed using the Illumina MiSeq platform as previously described [27]. The sequences were demultiplexed, quality filtered by the Quantitative Insights Into Microbial Ecology (QIIME, v1.8.0) software, and the Illumina reads were joined using the fastq-join method. Bacterial operational taxonomic unit (OTU) sequences were analyzed using the UCLUST algorithm with a 97% threshold of pairwise identity and classified taxonomically based on the Greengenes reference database. α-diversity was estimated by different microbial diversity metrics using QIIME software. β-diversity analysis, based on Bray–Curtic distance, was conducted using QIIME software and shown by the principal coordinates analysis (PCoA). Linear discriminant analysis (LDA) effect size (LEfSe) analysis was performed to identify differentially abundant bacterial taxa (from phylum to genus levels) on Galaxy website (http://huttenhower.sph.harvard.edu/galaxy/, accessed date: 9 August 2021) and the comparison of intestinal microbes was performed by the STAMP software.

Network was conducted to investigate co-occurrence patterns of bacterial taxa. The genera with relative abundances above 0.05% were selected. A Spearman’s correlation between two genera was considered statistically robust [28]. Each node in the correlation network stands for one genus, and each edge represents a significant correlation between the nodes. To determine the topology of the networks, a set of measures (average degree, graph density, modularity, positive correlation, negative correlation) were calculated using igraph packages in R environment [29]. The networks were visualized in the interactive platform Gephi [30]. Moreover, ErdÖs-Réyni random networks were used to compare with the topology of a real network [31].

### 2.8. Metagenomic Sequencing and Annotation

Metagenomic analysis was conducted to assess the functional and metabolic potential of the microbial communities. Samples were sequenced using the Illumina HiSeq2000 instrument (Illumina Inc., San Diego, CA, USA). Libraries were prepared with a fragment length of 300 bp. Paired-end reads were generated with 100 bp in the forward and reverse direction. The data of microbiota composition were analyzed using MetaPhlAn2. Metagenomic shotgun reads from each sample were searched against Kyoto Encyclopedia of Genes and Genomes (KEGG) gene database (version 58) using Mblastx and KEGG orthology (KO) system, to analyze the molecular interactions and reaction networks. The search results were run through HUMAnN2, a pipeline developed for obtaining pathway abundance and coverage from metagenomic communities. Heatmaps of microbial functions were conducted by R package.

### 2.9. Statistical Analysis

Data are presented as the mean ± standard deviation (SD). Statistical significance was determined by one-way ANOVA followed by Tukey test and two tailed Student’s *t*-test using SPSS 20.0 statistical software (SPSS Inc., Chicago, IL, USA). *p* < 0.05 indicated statistical significance. The co-occurrence network was conducted based on correlation analysis (Spearman’s R > 0.6, FDR-adjusted *p*-value < 0.05). Analyses for correlation assays were performed between biochemical parameters and microbes. Correlation coefficients were analyzed using Pearson’s correlation distance, and heatmaps were conducted by R package to identify bivariate relationships. Figures were prepared using Graphpad prism 8.0.

## 3. Results

### 3.1. Bacillus Attenuated Hepatic Injuries in DQ-Exposed Rats 

**Figure 1:**
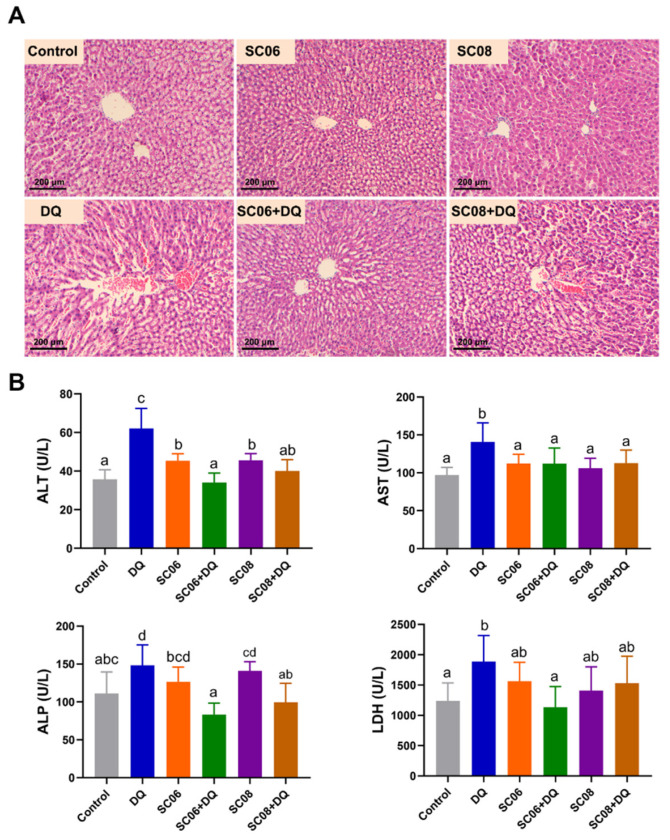
*Bacillus* attenuated hepatic injuries in diquat (DQ)-exposed rats. Rats were orally administered with 1 mL of 0.9% saline or 10^7^ cfu/mL *Bacillus* SC06 or SC08 for 24 days, and then intraperitoneally injected with 12 mg/kg BW DQ to induce oxidative stress. (**A**) Histomorphometric analysis of liver samples. Hematoxylin and eosin (H&E) staining, 100× magnification, scale bar: 200 μm. (**B**) Analysis of biochemical parameters of liver injuries. Data were analyzed by one-way ANOVA Tukey test (*n* = 8 in each group). Different letters (a–c) in each parameter represent significance (*p* < 0.05).

Results from H&E staining showed that liver injuries were obviously observed in DQ- treated rats, as evidenced by the disruption of integrated hepatic lobules, cytoplasmic degenerative changes and cellular vacuolization. However, SC06 decreased the tissue damages and reversed to the normal histological structure (Figure 1A). As shown in Figure 1B, compared with untreated rats, DQ significantly increased the levels of ALT, AST and ALP, which were markedly reduced by administration of *Bacillus* SC06 or SC08. SC06 administration significantly lowered the increased LDH level caused by DQ exposure. These results suggested that probiotics, particularly SC06 could ameliorate hepatic oxidative injuries.

### 3.2. Bacillus Decreased the Mitochondrial Dysfunction in DQ-Exposed Rats 

**Figure 2:**
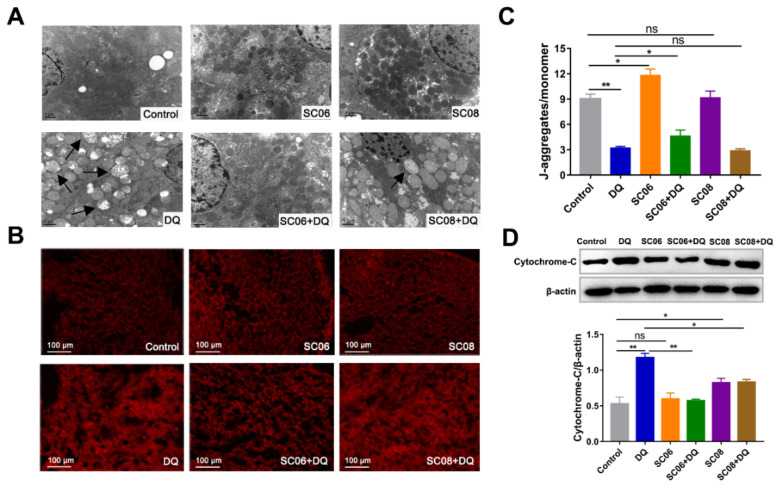
*Bacillus* decreased DQ-induced liver mitochondrial dysfunction. (**A**) TEM analysis of liver. Images were observed at 15,000× magnification, scale bar: 1 μm. (**B**) The detection of ROS production by DHE staining. 200× magnification, scale bar: 100 μm. (**C**) ΔΨm was calculated as the ratio of red fluorescence (JC-1 aggregates) to green fluorescence (JC-1 monomers). (**D**) Protein expression of the released cytoplasmic cytochrome-C. Cytochrome-C/β-actin was analyzed using Image J. Data were analyzed using one-way ANOVA with Tukey test. * *p* < 0.05; ** *p* < 0.01, ns = no significance.

The ultrastructure of liver mitochondria was observed by TEM. No obvious changes were found between probiotic-treated and control groups, but serious damages occurred in response to DQ exposure, as indicated by the enlarged and swollen shape, fuzzy and fractured ridge, vacuolation and transparent mitochondrial matrix (Figure 2A). However, probiotic administration, particularly SC06, attenuated the severity of the aberrant mitochondria. SC06 pretreatment could also significantly decreased ROS production (Figure 2B). ΔΨm loss is early evidence of the mitochondrial dysfunction. As shown in Figure 2C, a dramatic ΔΨm reduction was found in DQ-treated rats (*p* < 0.01) but was reversed by SC06 pretreatment (*p* < 0.05). ΔΨm loss is followed by cytochrome c release from the mitochondria to the cytoplasm. Results revealed that both probiotic pretreatments markedly inhibited the increased amount of cytoplasmic cytochrome c, compared to DQ group (*p* < 0.01 and *p* < 0.05, respectively) (Figure 2D).

### 3.3. Bacillus Enhanced the Antioxidant Capacity of Liver in DQ-Exposed Rats

**Figure 3:**
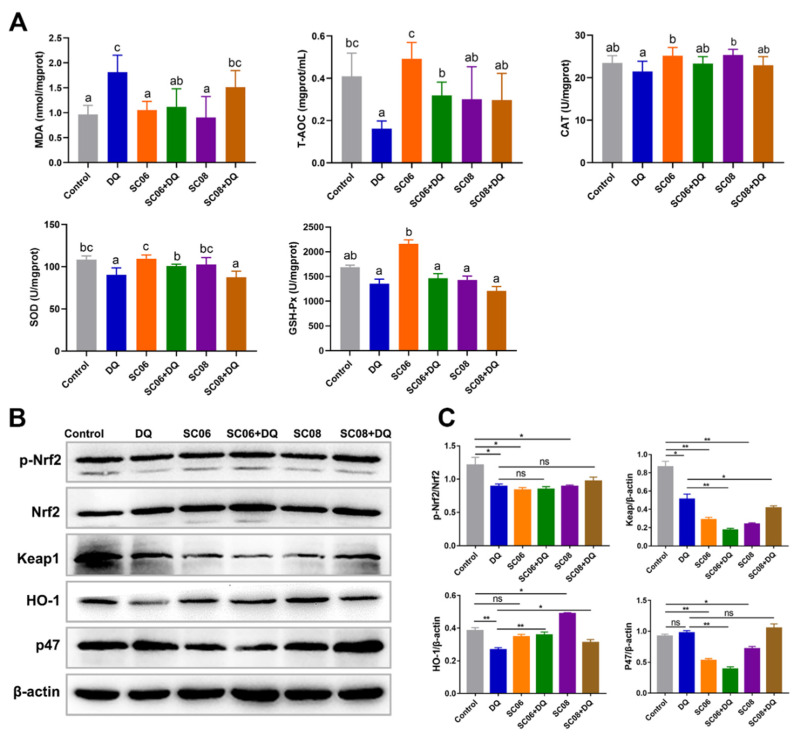
*Bacillus* enhanced the antioxidant capacity of liver in DQ-exposed rats. (**A**) Analysis of antioxidant parameters. Data were analyzed by one-way ANOVA Tukey test (*n* = 8 in each group). Different letters (a–c) in each parameter represent significant (*p* < 0.05). (**B**) The expression of antioxidant-related proteins. Liver lysates were collected to detect the protein expression of p-Nfr2, Nrf2, Keap1, HO-1 and p47. (**C**) Analyses of p-Nfr2, Nrf2, Keap1, HO-1 and p47 to β-actin using Image J (one-way ANOVA; The ratios were analyzed using ImageJ. Data were analyzed by one-way ANOVA Tukey test. * *p* < 0.05; ** *p* < 0.01, ns = no significance.

As displayed in Figure 3A, SC06 pretreatment markedly decreased MDA level induced by DQ exposure. DQ significantly reduced T-AOC, CAT and SOD, while the two probiotics increased T-AOC to varying degrees and exerted no effect to CAT. Only SC06 pretreatment increased SOD activity. No obvious changes were found in GSH-Px activity among all groups. We further detected the expression of proteins targeting antioxidant Keap1-Nrf2 signaling pathways. As shown in Figure 3B,C, probiotic treatments significantly degraded Keap1 (*p* < 0.05), but did not affect Nrf2 phosphorylation (*p* < 0.05). DQ exposure significantly decreased HO-1 expression (*p* < 0.01), but this trend was reversed by SC06 pretreatment (*p* < 0.01). Furthermore, SC06 markedly down-regulated the increased expression of NADPH oxidase subunit p47.

### 3.4. Bacillus Altered the Composition of Gut Microbiota

**Figure 4:**
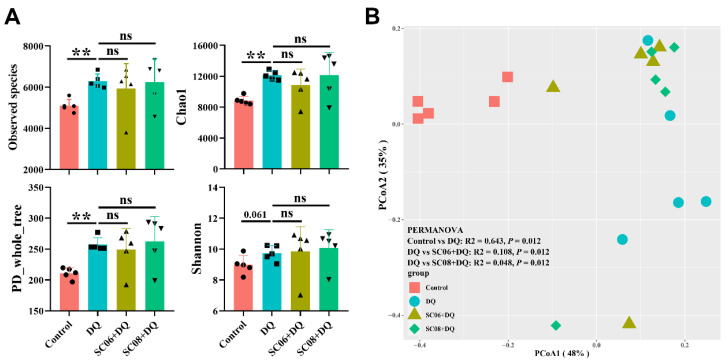
Analysis of the diversity of gut microbiota. (**A**) α diversity was reflected by Observed_species, Chao1, PD_whole_tree and Shannon indexes. Differences between groups were determined by one-way ANOVA followed by Tukey test (*n* = 5). ** *p* < 0.01, ns = no significance. (**B**) β-diversity was calculated by the Bray–Curtis distance and presented by principal coordinates analysis (PCoA) scatterplot. Significant differences between groups were analyzed by PERMANOVA.

We then targeted the gut microbiota to clarify the underlying mechanisms. DQ exposure significantly up-regulated the observed species, Chao1 and PD_whole_tree reflecting α-diversity (*p* < 0.01), and no obvious changes were found by probiotic treatments (*p* > 0.05) (Figure 4A). The Shannon index was not significant among the groups (*p* > 0.05). Bray_curtis was used to compare β-diversity between treatment groups. As shown in the PCoA scatterplot, DQ treatment caused a visible shift from the other groups (Figure 4B). Differences between DQ and probiotic- pretreated groups were found to be significant (PERMANOVA, *p* = 0.012).

**Figure 5:**
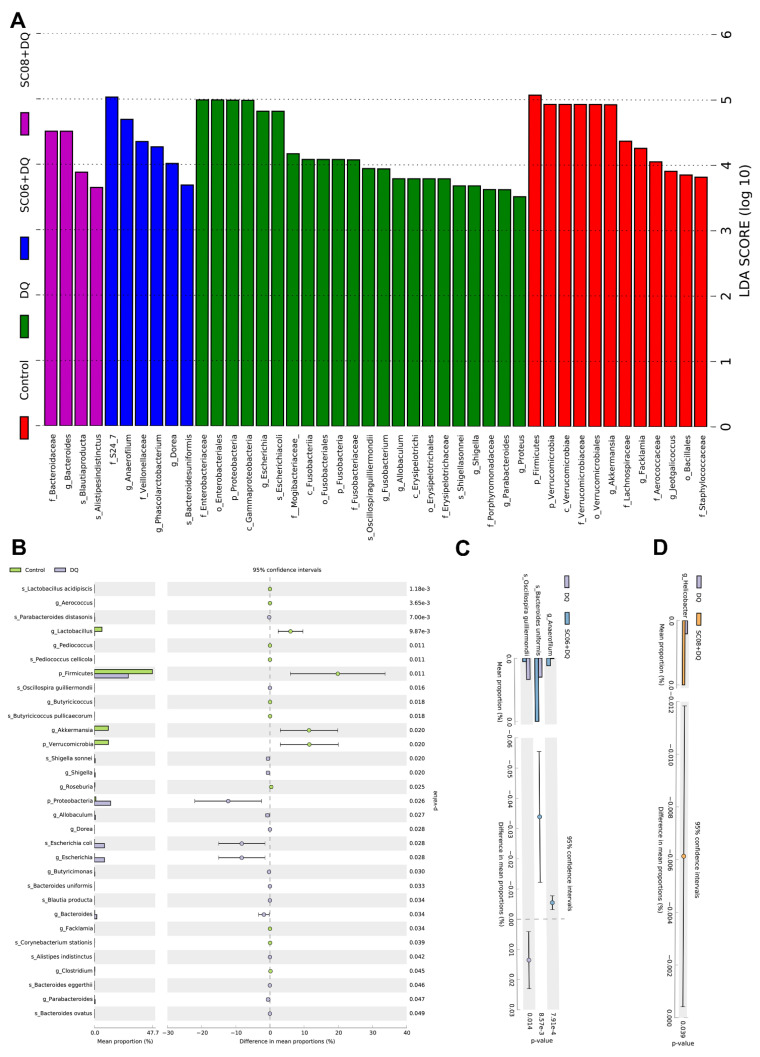
Analysis of the taxonomic biomarkers of gut microbiota. (**A**) Histogram of linear discriminant analysis (LDA) effect size (LEfSe) analysis. LDA score (log10) > 3 suggests dominant taxa in cases. (**B**–**D**) Comparison of gut microbes by statistical analysis of taxonomic and functional profiles (STAMP).

Results of LEfSe analysis were presented by a bar chart of six levels (from phylum to species) (Figure 5A). In the control group, Firmicutes presented the highest discriminative power (LDA score > 5). The phylum Verrucomicrobia, including c_Verrucomicrobiae, o_Verrucomicrobiales, f_Verrucomicrobiaceae and g_Akkermansia, were also enriched (LDA > 4, approximately 5). In DQ-exposed rats, the predominant bacteria belonged to the phylum Proteobacteria, i.e., c_gammaproteobacteria, o_*Enterobacteriales* f_Enterobacteriaceae, g_*Escherichia*, g_Shigella, s_*Escherichiacoli* and s_Shigellasonnei. Additionally, phylum Fusobacteria (c_Fusobacteriia, o_Fusobacteriales, f_Fusobacteriaceae and g_*Fusobacterium*) and the families *Erysipelotrichaceae* and *Porphyromonadaceae* also had a relatively large effect size (LDA score > 3.5). In SC06 + DQ group, the taxonomic biomarkers were f_S24_7, g_*Anaerofilum*, f_Veillonellaceae, g_*Phascolarctobacterium*, g_*Dorea* and s_*Bacteroidesuniformis*. The f_Bacteroidaceae, g_*Bacteroides*, s_*Blautiaproducta* and s_*Alistipesindistinctus* showed large effect sizes in SC08 + DQ group. As shown in Figure 5B–D, STAMP analysis revealed that compared to the control group, DQ exposure significantly decreased the abundance of g_*Lactobacillus*, p_Firmicutes, g_*Akkermansia* and p_Verrucomicrobia (*p* < 0.05), whereas markedly increased p_Proteobacteria, g_*Escherichia* and s_*Escherichiacoli* (*p* < 0.05). Compared to the DQ group, SC06 pretreatment significantly upregulated the levels of g_*Anaerofilum* and s_*Bacteroides* uniformis, and downregulated s_*Oscillospira guilliermondil* (*p* < 0.05). SC08 pretreatment markedly increased the richness of g_*Helicobacter* (*p* < 0.05). 

### 3.5. Analysis of Co-Occurrence Network and Pearson’s Correlation

**Figure 6:**
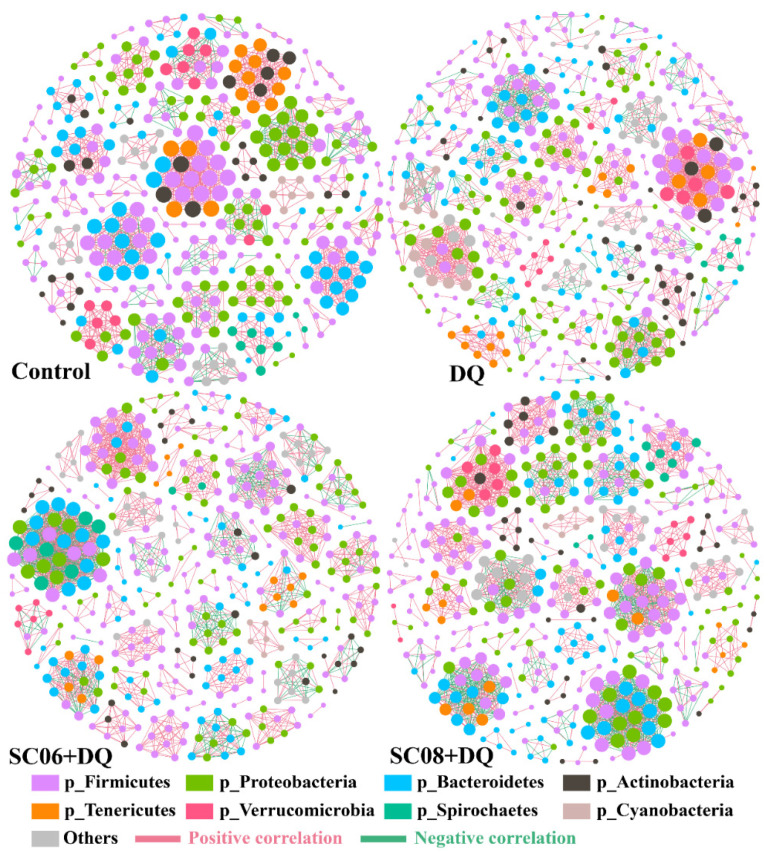
Co-occurrence networks of microbial communities based on correlation analysis. A connection stands for a very strong (Spearman’s R > 0.6) and significant (FDR-adjusted *p*-value < 0.05) correlation. The size of each node is proportional to the relative abundance; the thickness of each connection between two nodes (edge) is proportional to the value of Spearman’s correlation coefficients. Red lines indicate positive correlations and green lines represent negative correlations.

The co-occurrence network was based on the correlation analysis, which contained nodes (OTU levels) and edges (Table 1 and Figure 6). Results revealed that the average degree and graph density in the DQ group were lower than Control, SC06 + DQ and SC08 + DQ groups. The modularity values in all groups were >0.4, and *Bacillus* pretreatments downregulated it. The positive correlation of microbial community was increased by DQ treatment but decreased by SC06 and SC08 pretreatments. On the contrary, DQ treatment decreased the negative correlation, whereas probiotic administration increased it. 

**Figure 7:**
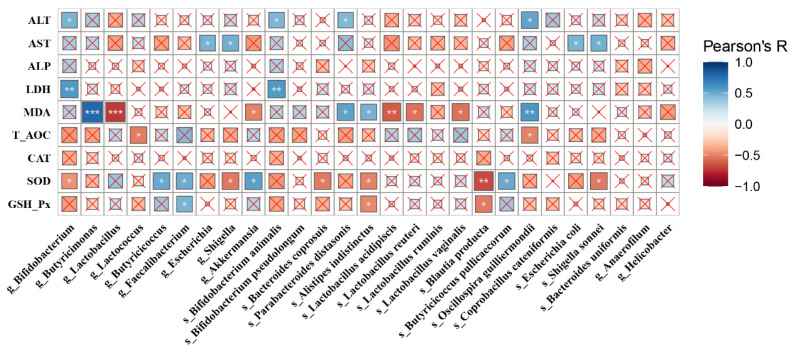
A heatmap of Pearson’s correlation. Correlation between the gut microbes and biochemical parameters (ALT, AST, ALP, LDH, MDA, T-AOC, CAT, SOD and GSH-Px). The sizes of boxes reflect the correlation coefficient. The depth of colors was according to the Pearson’s correlation coefficient distribution. ^×^ *p* > 0.05, * *p* < 0.05, ** *p* < 0.01, *** *p* < 0.001.

We then examined the extent to which the antioxidant functions of *Bacillus* would correlate with microbiota alterations. Pearson’s correlation was used to analyze the significant genera and species with the indices (ALT, AST, ALP, LDH, MDA, T-AOC, CAT, SOD and GSH-Px) (Figure 7). Results revealed that ALT showed a significantly positive correlation with g_*Bifidobacterium*, s_*Bifidobacterium animalis,* s_*Parabacteroides distasonis* and s_*Oscillospira guilliermondii* (*p* < 0.05)*,* while AST was positively linked with g_*Escherichia*, g_*Shigella*, s_*Escherichia coli* and s_*Shigella sonnei* (*p* < 0.05). MDA exhibited a highly positive correlation with g_*Butyricimonas* (*p* < 0.001), s_*Oscillospira guilliermondii* (*p* < 0.01), s_*Parabacteroides distasonis* (*p* < 0.01) and s_*Alistipes indistinctus* (*p* < 0.01), and a negative correlation with g_*Lactococcus* (*p* < 0.001), g_*Akkermansia* (*p* < 0.05), s_*Lactobacillus acidipiscis* (*p* < 0.01), s_*Lactobacillus reuteri* (*p* < 0.05), and s_*Lactobacillus vaginalis* (*p* < 0.05). SOD had a highly positive correlation with g_*Butyricicoccus*, g_*Faecalibacterium*, g_*Akkermansia* and s_*Butyricicoccus pullicaecorum* (*p* < 0.05), and a negative correlation with g_*Bifidobacterium*, g_*Shigella,* s_*Bacteroides coprosuis*, s_*Alistipes indistinctus*, s_*Blautia producta* (*p* < 0.001) and s_*Shigella sonnei* (*p* < 0.05). Similar trends were also found in GSH-Px.

### 3.6. Bacillus Suppressed the Microbial Metabolic Signaling Pathways 

**Figure 8:**
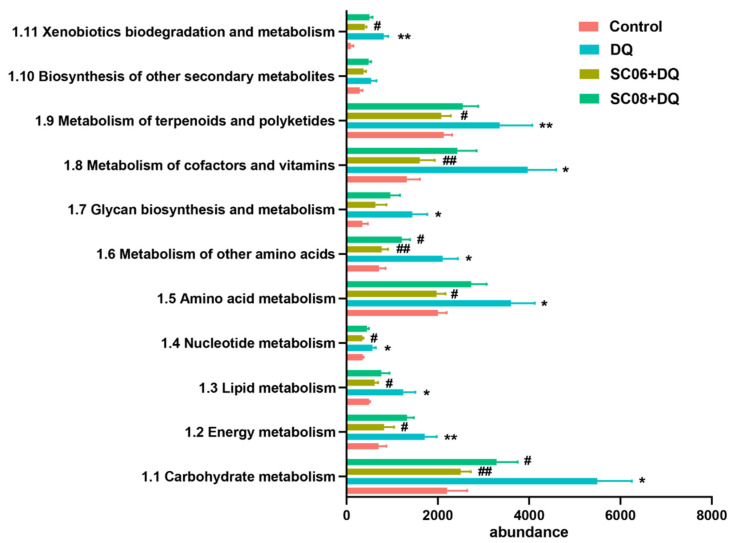
Relative abundance of the metabolic functional genes of gut microbiota. The metabolic pathways were analyzed using Mblastx and the KEGG orthology (KO) system. Data were shown at the second level of KEGG pathways. * *p* < 0.05; ** *p* < 0.01. ^#^ *p* < 0.05; ^##^ *p* < 0.01.

The metagenomics analysis was performed to explore the effects of probiotics on microbiome functionality during oxidative stress. At the first level of KEGG pathways, DQ exposure showed a marked increase in the function of gut microbial metabolism while *Bacillus* pretreatments, particularly SC06 down-regulated the increased trend (data not shown). Results from the KEGG second level involving metabolism showed that DQ exposure activated all the metabolic pathways, whereas *Bacillus* administrations could reverse this trend. Specifically, the gene abundance for pathways such as carbohydrate metabolism, energy metabolism, lipid metabolism, nucleotide metabolism, amino acid metabolism, metabolism of cofactors and vitamins, and metabolism of terpenoids and polyketides were markedly upregulated by DQ exposure, but significantly downregulated by *Bacillus* pretreatments, particularly SC06 (*p* < 0.05) (Figure 8). 

**Figure 9:**
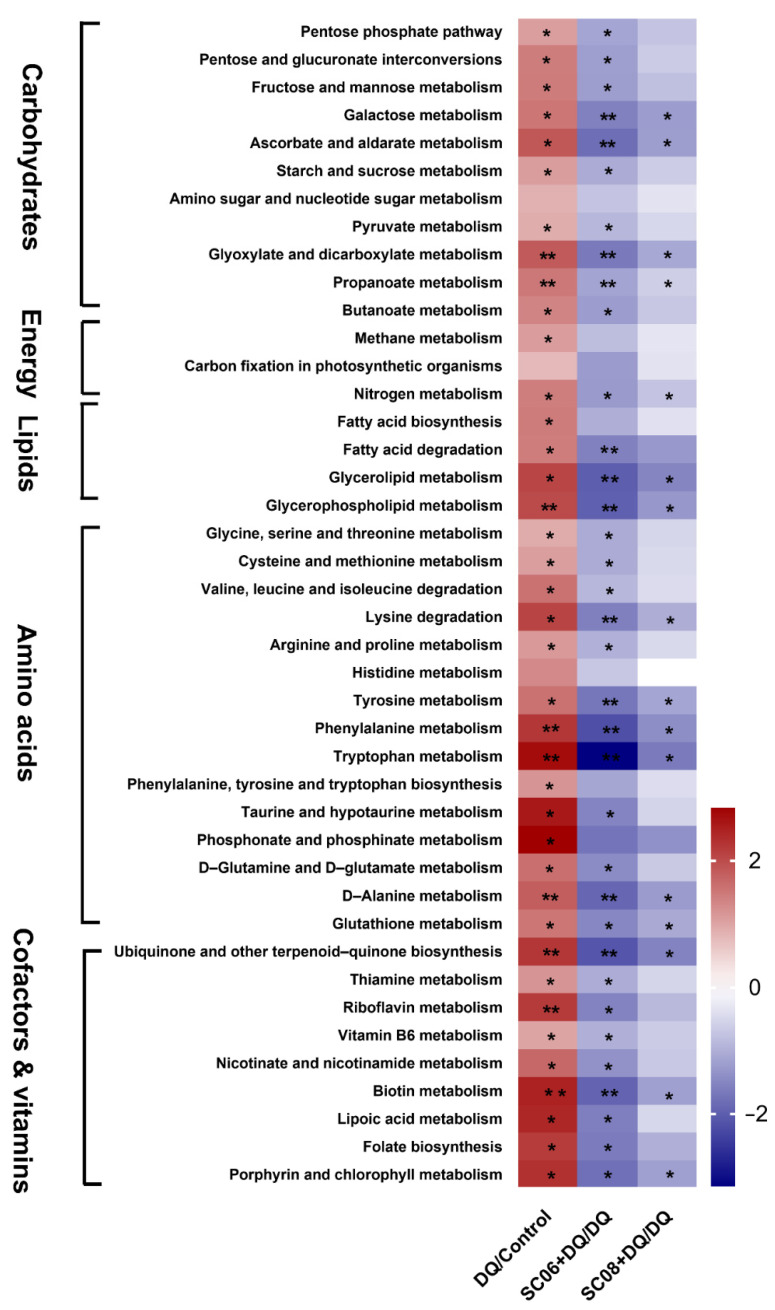
The significant metabolic pathways of gut microbiota. Differences in metabolic pathways were analyzed using Mblastx and the KEGG orthology (KO) system. The presented metabolic pathways were screened by the fold change > 2 or fold change < 0.5 using the formula DQ/Control, (SC06 + DQ)/DQ and (SC08 + DQ)/DQ. The heatmap was performed using the fold-change value (log2 transformed) of the gene abundance of metabolic pathways. * *p* < 0.05; ** *p* < 0.01.

We then further analyzed the specific metabolic pathways by functional annotation of the KO system. As shown in Figure 9, DQ treatment markedly increased pathways involving carbohydrate metabolism, i.e., Pentose phosphate pathway, Pentose and glucuronate interconversions, Fructose and mannose metabolism, Galactose metabolism, Starch and sucrose metabolism, pyruvate metabolism, Glyoxylate and dicarboxylate metabolism, Propanoate metabolism and Butanoate metabolism (*p* < 0.05), while *Bacillus* pretreatments, particularly SC06 could significantly reverse this trend (*p* < 0.05). Moreover, *Bacillus* inhibited the gene abundance of nitrogen metabolism pathway (*p* < 0.05). A dramatic downregulation of lipid metabolism pathways such as fatty acid degradation, Glycerolipid metabolism and Glycerophospholipid metabolism were found in SC06 pretreatment (*p* < 0.01). DQ exposure significantly increased the gene enrichment of amino acid metabolism, while *Bacillus* administration, particularly SC06, showed a strong ability to suppress the upregulated levels (*p* < 0.05). Specifically, SC06 dramatically reduced the genes involving in Lysine degradation, Tyrosine metabolism, Phenylalanine metabolism, Tryptophan metabolism and D-alanine metabolism (*p* < 0.01). Results also revealed an obvious inhibition of metabolism of cofactors and vitamins by SC06 pretreatments, especially the pathways Ubiquinone and other terpenoid-quinone biosynthesis and Biotin metabolism (*p* < 0.01). All the above results indicated that *Bacillus* pretreatment showed a strong capacity to suppress the microbial metabolic pathways that was upregulated by DQ exposure.

### 3.7. Bacillus Inhibited the Pathways Mediating Bacterial Replication and Secretion 

**Figure 10:**
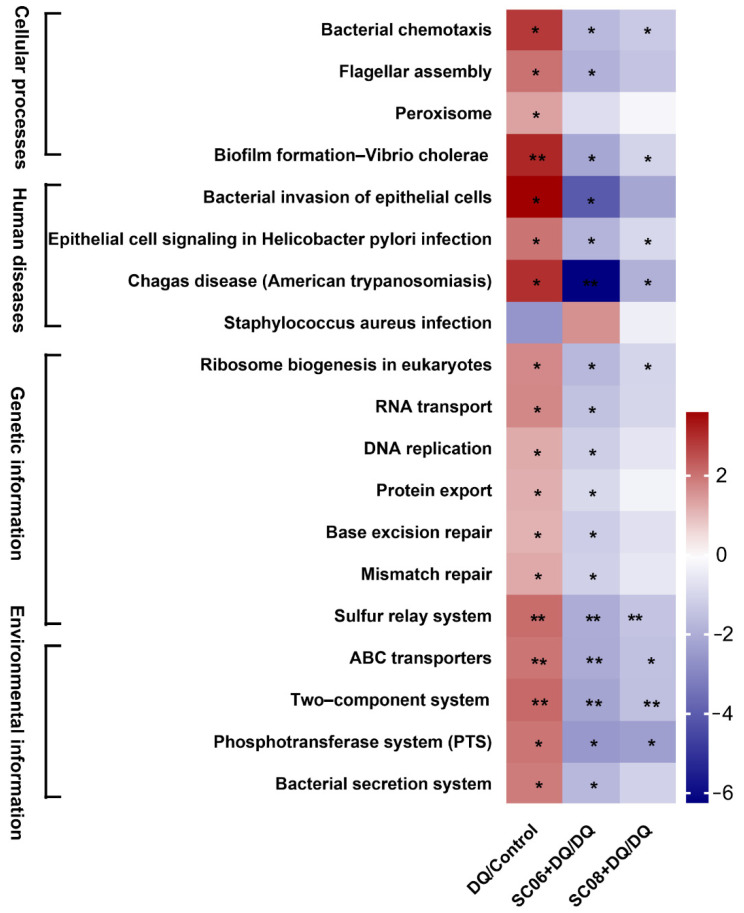
Analysis of pathways mediating bacterial replication and secretion. Data were analyzed using Mblastx and the KEGG orthology (KO) system. The presented pathways were screened by the fold change > 2 or fold change < 0.5 using the formula DQ/Control, (SC06 + DQ)/DQ and (SC08 + DQ)/DQ. The heatmap was performed using the fold-change value (log2 transformed) of the gene abundance of metabolic pathways. * *p* < 0.05; ** *p* < 0.01.

As displayed in Figure 10, DQ significantly increased the KEGG pathways at the first level, i.e., cellular processes, human diseases, genetic information and environmental information, whereas *Bacillus* pretreatments, particularly SC06 could reverse this trend. To be specific, SC06 markedly downregulated the gene abundance of the pathways involving bacterial chemotaxis, flagellar assembly, biofilm formation, bacterial invasion of epithelial cells, epithelial cell signaling in Helicobacter pylori infection, Chagas disease, ribosome biogenesis in eukaryotes, RNA transport, DNA replication, protein export, base excision repair and mismatch repair (*p* < 0.01), which are mainly involved in the replication, secretion and pathogenicity of pathogens.

## 4. Discussion

The potential of *Bacillus* as a probiotic has gained more and more scientific interest in recent years. Compared with the commonly explored probiotic strains, such as lactic acid bacteria, *Bacillus* is a genus of endospore-forming bacteria which allows extreme temperature tolerance and longer storage without viability loss; also, spores can survive very low pH of stomach and completely reach the small intestine [32]. This advantage makes them more suitable candidates for pharmaceutical application and food supplements. Recent studies showed that some *Bacillus* strains, for example, *B. amyloliquefaciens*, *B. methylotrophicus* and *B. subtilis* exhibit potent antioxidant capacity to alleviate oxidative injuries [33,34,35]. The current study revealed the antioxidant functions of two *Bacillus* species, *B. amyloliquefaciens* SC06 and *B. licheniformis* SC08 in preventing liver injuries. Similar with our results, the alleviation of liver injury by *Bacillus* has been confirmed by many studies. For instance, *Bacillus* spores protected against acetaminophen-induced acute liver injury in rats [36]. Another study found that *B. amyloliquefaciens* B10 alleviated aflatoxin B1-induced liver apoptosis and oxidative stress, and it showed that B10 could ameliorate the irregularly arranged hepatocytes and their damaged areas [37]. Hepatic stellate cells are a key player in the progression of liver fibrosis and can be activated by various stimuli including oxidative stress [38]. Recent research has showed that probiotics such as *Akkermansia muciniphila* and *Lactobacillus* could activate stellate cells to inhibit liver fibrosis [39,40]. Whether *Bacillus* SC06 or SC08 modulates the functions of stellate cells to alleviate oxidative stress warrants further investigation. We also found that *Bacillus* SC06 showed stronger antioxidant capacity than SC08, as evidenced by the ameliorated hepatic injuries, decreased mitochondrial dysfunction and enhanced antioxidant levels under DQ exposure. Consistent with our previous findings [26], these results further confirmed that probiotics are highly species- or even strain-specific [41]. 

ROS can be eliminated by several antioxidant systems, including the activation of Keap1/Nrf2 signaling and NAD^+^-dependent SIRT_4_ activity, and the production of antioxidant enzymes [42]. Oxidative stress inactivates Keap1 which leads to Nrf2 release and translocation into the nucleus, and finally activates the expression of antioxidant genes [43]. Our results showed that *Bacillus* treatments significantly degraded Keap1 but did not affect Nrf2 phosphorylation. NAD^+^-dependent SIRT_4_ has been reported to be a key regulator of metabolic enzymes and antioxidant defense mechanisms, particularly in regulating mitochondrial metabolism [44]. On the contrary, another report revealed that SIRT_4_ modulation of fatty acid metabolism reduced the levels of free fatty acids but unfortunately increased ROS production in obese patients with NAFLD [45]. In our study, *Bacillus* SC06 significantly decreased the mitochondrial dysfunction in DQ-exposed rats, but whether SIRT_4_ participated in this process was not investigated. Nevertheless, another study in our lab showed that SC06 could activate SIRT_1_/FOXO_3_ signaling to alleviate oxidative stress in IPEC-J2 cells [46]. HO-1 is a primary antioxidase that exerts anti-oxidative injuries and antiapoptotic functions [47]. We found that *Bacillus* showed the capacity to reverse the decreased level of HO-1 by DQ exposure. All the findings demonstrated that SC06 could modulate the defensive system upon oxidative stress.

By using 16S rRNA sequencing analysis, we demonstrated that *Bacillus* SC06 protected the liver against oxidative stress by altering gut microbiota. Evidence suggested that modulation of gut microbiota have the potential to control hepatic cellular stress and treat liver diseases of different etiologies [48,49]. In this study, 16S rRNA analysis revealed that DQ exposure led to the dysbiosis of microbial composition by decreasing the proportion of Firmicutes and increasing Proteobacteria, whereas probiotic pretreatments could reverse this trend. The Gram-positive Firmicutes is the dominant bacterial phylum in the healthy state in humans. It includes the genera *Lactobacillus*, *Clostridium*, *Ruminococcus* and butyrate producers *Faecalibacterium* and *Eubacterium* [50]. *Proteobacteria* phylum contains most of the opportunistic pathogens, including *Escherichia*, *Salmonella*, *Helicobacter* and *Vibrio* [51]. Accumulating studies have revealed the expansion of *Proteobacteria* in many liver diseases, such as the advanced fibrosis of NAFLD, hepatitis B liver cirrhosis and NASH [52,53,54]. One study even confirmed the fact that fecal transplantation of *Proteobacteria* resulted in a significant increase in cholestatic liver fibrosis when compared with mice transplanted with Gram-positive bacteria [55]. In our study, DQ exposure markedly reduced the proportion of Firmicutes and *Lactobacillus*, and increased Proteobacteria, while probiotic administration could attenuate this reduction, suggesting the ability of probiotics in optimizing gut balance during oxidative stress. Similar to our findings, in a mouse model of ALD, probiotic LGG treatment increased the level of *Firmicutes* and prevented ethanol-induced upregulation of *Proteobacteria* [56]; probiotics could reduce the *Proteobacteria* population in multiple liver disease in rats [57,58]. 

Intestine anaerobiosis is believed to drive the composition of microbiota towards a dominance of obligate anaerobes, while as a facultative anaerobe, the upregulation of *Proteobacteria* indicates a disruption in anaerobiosis, which may cause epithelial dysfunction and the pathogenesis of diseases [59]. By STAMP analyses, we observed a significantly increase in the abundance of opportunistic pathogens p_Proteobacteria, g_*Escherichia* and s_ *Escherichia coli*, and a decrease in the potential probiotics g_*Lactobacillus*, p_Firmicutes, g_*Akkermansia* and p_Verrucomicrobia in the DQ group, indicating the gut dysbiosis caused by oxidative stress. Compared to the DQ group, SC06 pretreatment markedly upregulated the richness of g_*Anaerofilum*, s_*Bacteroides uniformis* and downregulated s_*Oscillospira guilliermondii*. *Anaerofilum* is a genus belonging to f_Ruminococcaceae that shows a superior capacity to degrade fiber and produce metabolites that are beneficial to the host. It was reported that *Anaerofilum* can produce a range of fermented products including lactate, acetate, formate and ethanol [60]. *Bacteroides uniformis* is regarded as a beneficial microbe that can utilize oligo- and polysaccharides to produce short-chain fatty acids [61]. Studies have confirmed its protective role in ameliorating metabolic and immunological dysfunction [62,63]. *Oscillospira* has dual functions for the host, as it positively associates with leanness and health, but also as an indicator for some diseases such as constipation and gallstones [64]. In our study, the decreased *Oscillospira* indicated the alleviated oxidative stress. Therefore, our findings suggest that SC06 could optimize the composition of gut microbiota and restore the gut dysbiosis induced by DQ exposure. Co-occurrence networks provide insight into microbial interactions, and we found that the average degree and graph density of microbial network in *Bacillus* groups were higher than those of the other groups, suggesting that *Bacillus* pretreatments increased microbe connections. The modularity values in all groups were >0.4, indicating a modular structure of the network [65]. Furthermore, positive correlation of the microbial networks in *Bacillus* groups was lower than that of DQ group, suggesting a reduction in competitive relationships within gut microbes [66]. Results of Pearson’s correlation showed that the beneficial microbes such as *Lactobacillus*, *Faecalibacterium* and *Butyricicoccus* exhibited a negative correlation with MDA, whereas g_*Escherichia* and g_*Shigella* were positively correlated with ALT and AST, which further confirmed that the alteration of microbiota composition was related to attenuating oxidative stress-induced liver injuries.

The functional KEGG annotation analyses of metagenomic study indicated that *Bacillus* administration, particularly SC06 could inhibit the microbial metabolic pathways including carbohydrate metabolism, lipid metabolism, amino acid metabolism and metabolism of cofactors and vitamins). SC06 pretreatment markedly decreased the gene enrichment of carbohydrates such as galactose metabolism. Accumulating studies have revealed that galactose plays a pivotal role in oxidative stress, cognitive impairment and mitochondrial dysfunction [67,68]. Furthermore, galactose is essential for the synthesis of various exopolysaccharides including colanic acid in *E. coli* [69]. Thus, the decreased levels of galactose might contribute to SC06-meditated suppression of pathogenetic bacteria growth and oxidative stress. Moreover, SC06 could decrease amino acid metabolism, particularly for phenylalanine and tryptophan. Phenylalanine metabolism provides substrate molecules for citric acid cycle and glycolysis, such as succinyl-CoA, Acetyl-CoA, fumarate, pyruvate and succinate. It was believed that the citric acid cycle operates only in aerobic organisms [70]. The inhibited activity of the citric acid cycle by SC06 suggested the lower composition of aerobic bacteria. Furthermore, Kohanski et al. identified citrate cycle-dependent upregulation of respiration as a significant source of antibiotic-induced oxidative stress, which revealed a complicated link between citrate cycle and ROS homeostasis [71]. Tryptophan metabolism was recognized as an important participant in immune response, inflammation and oxidative stress [72]. Studies also found that tryptophan takes an important part in the biofilm formation of pathogens such as *Salmonella* Typhimurium; deletion of tryptophan genes led to the decreased bacterial attachment and biofilm defect [73]. Therefore, the downregulation of phenylalanine and tryptophan mechanisms indicated a decreased level of oxidative stress and pathogen growth by SC06 pretreatment. In addition, DQ exposure significantly promoted the biosynthesis and metabolism of various B vitamins (e.g., Thiamine, Riboflavin, Vitamin B6, Biotin, Lipoic acid and Folate) of gut microbiota, while SC06 pretreatment significantly downregulated them. B vitamins are essential in many metabolism processes including carbohydrate, amino acid, lipid metabolisms and DNA synthesis. It was reported that B vitamins play an important role upon oxidative stress. For instance, Van De Lagemaat found that B Vitamins scavenge reactive oxygen species and modulate immune cytokines to reduce oxidative stress [74]; supplementation with B vitamins enhanced the anti-oxidative state in patients with liver cancer [75]. Inconsistently, we found that the genes for vitamin B pathways were more abundant in DQ group but markedly decreased in *Bacillus*-treated groups. The underlying mechanisms might be that DQ challenge led to a compensatory and accelerated metabolism of B vitamins to protect against acute oxidative damages. However, *Bacillus* pretreatment exerted strong anti-oxidant capacity and recovered to normal physiological status in the organism, hence exhibiting a suppressed metabolism.

We further demonstrated that *Bacillus* SC06 inhibited the pathways mediating bacterial assembly, secretion and pathogenicity. SC06 significantly decreased the gene enrichment involved in cellular processes such as bacterial chemotaxis, flagellar assembly and biofilm formation. Bacteria use chemotaxis to detect various environmental stimuli and migrate towards environments that are favorable for growth and survival [76]. It was reported that pathogens such as *E. coli* can utilize chemotaxis towards host-derived chemicals or self-produced signaling molecules for bacterial aggregation and biofilm formation [77]. The bacterial flagellum, particularly highly developed in Gram-negative bacteria, is a supramolecular motility apparatus that consists of the basal body, the hook, and the filament [78]. It allows bacteria to migrate toward favorable conditions and escape from undesirable ones; it can also respond to environmental stimuli by inducing developmental changes such as cell differentiation and biofilm formation for their survival [79]. Biofilm formation is regarded as one of the primary virulence factors that allows microorganisms to absorb nutrients and withstand hostile environments [80]. It was reported that the potential capacity of probiotics to inhibit biofilm formation represents a strategy for reducing microbial colonization on epithelial mucosa that subsequently leads to infections [81]. In our study, SC06 significantly downregulated the expression of the key genes involved in the pathways for bacterial chemotaxis, flagella assembly and biofilm formation, suggesting its potential ability to inhibit pathogen growth and colonization. Results also revealed that SC06 pretreatment decreased the gene enrichment of bacterial secretion systems. The bacterial secretion system is one of the essential components for many bacterial pathogens to invade mammalian hosts. Secreted proteins are pivotal in promoting bacterial virulence, such as enhancing attachment to host cells, scavenging resources in an environmental niche, and directly intoxicating target cells to disrupt their functions [82]. These results confirmed SC06 administration downregulated the pathways associated with the pathogenicity of opportunistic pathogens, and exerted a protective role in alleviating oxidative stress- induced microbiota imbalance.

## 5. Conclusions

In conclusion, *Bacillus* SC06 alleviated oxidative stress-induced live injuries via modulating the composition, and pathways for metabolism and bacterial replication and secretion of gut microbiota. Our findings elucidate the mechanisms of probiotics in alleviating oxidative stress and provide a promising strategy for preventing liver disease by targeting the gut microbiota.

## Figures and Tables

**Table 1:** Topological properties of co-occurrence network.

	Control	DQ	SC06 + DQ	SC08 + DQ
Nodes	404	457	439	440
Edges	1658	1812	2121	2122
Average degree (AD)	8.208	7.93	9.663	9.645
Graph density (GD)	0.02	0.017	0.022	0.022
Modularity (MD)	0.951	0.941	0.918	0.934
Positive correlation	81.97%	82.62%	74.35%	71.77%
Negative correlation	18.03%	17.38%	25.65%	28.23%

## Data Availability

Raw sequences have been deposited in the Genome Sequence Archive (GSA) of the BIG Data Center (https://bigd.big.ac.cn/gsa/, accessed date: 21 October 2021) under accession number PRJCA006927/CRA005217.
